# Carnosine Reduces Oxidative Stress and Reverses Attenuation of Righting and Postural Reflexes in Rats with Thioacetamide-Induced Liver Failure

**DOI:** 10.1007/s11064-015-1821-9

**Published:** 2016-01-22

**Authors:** Krzysztof Milewski, Wojciech Hilgier, Inez Fręśko, Rafał Polowy, Anna Podsiadłowska, Ewa Zołocińska, Aneta W. Grymanowska, Robert K. Filipkowski, Jan Albrecht, Magdalena Zielińska

**Affiliations:** Department of Neurotoxicology, Mossakowski Medical Research Centre, Polish Academy of Sciences, Pawińskiego 5 Str, 02-106 Warsaw, Poland; Behavior and Metabolism Research Laboratory, Mossakowski Medical Research Centre, Polish Academy of Sciences, Pawińskiego 5 Str, 02-106 Warsaw, Poland

**Keywords:** Hepatic encephalopathy, Oxidative stress, Neuroprotection, Histidine, Carnosine, Righting reflex, Postural reflex

## Abstract

Cerebral oxidative stress (OS) contributes to the pathogenesis of hepatic encephalopathy (HE). Existing evidence suggests that systemic administration of l-histidine (His) attenuates OS in brain of HE animal models, but the underlying mechanism is complex and not sufficiently understood. Here we tested the hypothesis that dipeptide carnosine (β-alanyl-l-histidine, Car) may be neuroprotective in thioacetamide (TAA)-induced liver failure in rats and that, being His metabolite, may mediate the well documented anti-OS activity of His. Amino acids [His or Car (100 mg/kg)] were administrated 2 h before TAA (i.p., 300 mg/kg 3× in 24 h intervals) injection into Sprague–Dawley rats. The animals were thus tested for: (i) brain prefrontal cortex and blood contents of Car and His, (ii) amount of reactive oxygen species (ROS), total antioxidant capacity (TAC), GSSG/GSH ratio and thioredoxin reductase (TRx) activity, and (iii) behavioral changes (several models were used, i.e. tests for reflexes, open field, grip test, Rotarod). Brain level of Car was reduced in TAA rats, and His administration significantly elevated Car levels in control and TAA rats. Car partly attenuated TAA-induced ROS production and reduced GSH/GSSG ratio, whereas the increase of TRx activity in TAA brain was not significantly modulated by Car. Further, Car improved TAA-affected behavioral functions in rats, as was shown by the tests of righting and postural reflexes. Collectively, the results support the hypothesis that (i) Car may be added to the list of neuroprotective compounds of therapeutic potential on HE and that (ii) Car mediates at least a portion of the OS-attenuating activity of His in the setting of TAA-induced liver failure.

## Introduction

Acute or chronic liver failure leads to the development of HE [1] which is currently defined as a complex neuropsychiatric syndrome in which an increase of blood and brain ammonia concentration seems to be a main causative factor [2–4]. In both humans suffering from HE and animals with induced HE, brain edema is a dominating fatal consequence of elevated ammonia and other pathogens involved (e.g. inflammation, hyponatremia, hemorrhage, etc.) [5, 6]. It is well established that OS ensuing excessive generation of reactive oxygen species, is a major contributor to brain edema and other pathogenic events in HE or hyperammonemia [7–9].

Carnosine (β-alanyl-l-histidine, Car) is present in various organs and tissues including the central nervous system, and can easily enter the brain from the periphery [10]. There are reasons to assume that cell protective potential of Car relies on its antioxidant activity, mediated by different mechanisms involving metal chelation, scavenging ROS and/or peroxyl radicals. Indeed, Car has a well-documented neuroprotective action against OS associated with cerebral ischemia, stroke or subarachnoid hemorrhage [11–13] and has been ascribed a wide range of cells protective properties in peripheral tissues [14]. With regard to the issue of liver failure, Car has been reported to prevent TAA- and carbon tetrachloride-induced liver injury in rats [15, 16]. Here we tested the hypothesis that Car may be likewise neuroprotective in TAA-induced liver failure. The brain OS parameters studied included: reactive oxygen species (ROS), total antioxidant capacity (TAC), GSH/GSSG ratio and thioredoxin reductase (TRx) activity. We also carried behavioral tests, analyzing cognitive and motor functions which are typically impaired in the setting of HE [17, 18].

Car remains in a reversible precursor-product relationship with His [for a review see [19], which prompted us to speculate that in the in vivo setting, administration of one or the other may evoke similar effects on brain metabolism and function. With regard to His, there is an evidence to suggest that its systemic administration may be a potentially useful treatment modality for the brain edema associated with HE [20]. The effect of His was primarily ascribed to the inhibition of excessive l-glutamine transport to mitochondria (the “Trojan Horse” hypothesis) [21, 22]. However, His is also known for its antioxidant action [23, 24]. Moreover, studies from our laboratory have shown that His also prevents reduction of glutathione content in brain mitochondria of TAA rats [25] and increases the total antioxidant capacity (TAC) of TAA rat brain [26]. In the same TAA model of HE, intraperitoneal (i.p.) administration of His lowered brain concentration of asymmetric dimethyl arginine (ADMA), an endogenous nitric oxide synthases (NOS) inhibitor and increased total activity of NOS, as was analyzed in brain homogenates [27]. The measurement of His concentration in brain homogenates from rats administered with His, revealed no significant increase of its content, despite well-evidenced protective effects [27]. Thus, we assumed that at least some of the protective effects of His administration on parameters connected with OS in TAA-induced HE in rats, may be due to the action of Car. Specifically, Car as a His metabolite, may contribute to the anti-OS activity of His in this condition.

## Materials and Methods

### Materials

The primary antibody CARNS-1 were from Proteintech Group, Inc., USA. carboxy-H_2_DCFDA was from Life-Technologies, USA. Total Antioxidant Capacity kit was from Cell Biolabs, USA. Secondary GAPDH antibody, thioacetamide, histidine, carnosine and all other chemicals and kits were bought from Sigma-Aldrich Chemical Co. (Steinheim, Germany).

### HE Model

Adult male Sprague–Dawley rats, weighing 250–280 g were housed in temperature and light controlled housing and given free access to food and water. All experiments were performed with agreement of local animal ethical committee that approved the experimental design. ALF was induced by three intraperitoneal (i.p.) injections of thioacetamide (TAA); 300 mg/kg body weight, at 24 h intervals [28]. His or Car at a dose of 100 mg/kg was injected i.p., 2 h before each TAA administration. Control rats were analogically injected with sodium saline solution. Animals were sacrificed 24 h after the last injection together with the onset of acute HE symptoms, including elevated blood and brain ammonia level. Isolated brain tissue (prefrontal cortex) was rapidly homogenized in a buffer appropriate to each protocol or frozen at −80 °C.

### HPLC Analysis

The brain cortex and plasma His and β-Ala (not shown) concentration were analyzed using HPLC with fluorescence detection after derivatization in a timed reaction with *o*-phthalaldehyde plus mercaptoethanol, as described earlier [27]. Samples (50 μL) were injected onto 150 × 4.6 mm 5 μm Hypersil ODS column, eluted with a mobile phase of 0.075 M KH_2_PO_4_ solution containing 10 % (v/v) methanol, pH 6.2 (solvent A), and methanol (solvent B). The methanol gradient was 20–70 % and the elution time was 20 min. Carnosine (Car) was separated using a mobile phase of 0.3 M sodium acetate with methanol (3/1, v/v) pH 5.5 and row rate 1.5 ml/min, as described by [29].

### Total Antioxidant Capacity

Total antioxidant capacity (TAC) was determined using an OxiSelect™ Total Antioxidant Capacity Assay Kit (Cell Biolabs, USA). Fresh tissue was homogenized (1/5; w/v) in Assay Buffer, centrifuged (12,000×*g*, 15 min, 4 °C) and supernatant was used to analysis of TAC and protein level. The TAC Assay based on the reduction of copper (II) to copper (I) by antioxidants. Approx. 30 µg of protein was used in reaction mixture, after reduction the copper (I) ion reacts with a coupling chromogenic reagent that produces a color detected spectrophotometrically at 490 nm. The net absorbance values were compared with a known uric acid standard curve. Results were expressed as “mM Uric Acid Equivalents/mg of protein”.

### Thioredoxin Reductase Activity

Thioredoxin Reductase (TrxR) activity was measured using a Thioredoxin Reductase Assay Kit (Sigma-Aldrich, Germany) according to the manufacturer’s protocol. Tissue was homogenized (1/5; w/v), centrifuged (12,000×*g*, 5 min, 4 °C) and supernatant was used for analysis of enzyme activity and protein determination. Protein extract was used in a reaction mixture, addition of 5,5-dithiobis-(2-nitrobenzoic acid) (DTNB) solution initiated the reaction and the change in absorbency caused by 2-nitro-5-thiobenzoic acid (TNB) production was measured after 1 min at 415 nm. The results are presented as nmol TNB/min/mg of protein.

### Reactive Oxygen Species Determination

The total content of the ROS species was determined with fluorimeter using a fluorescent probe, 5-(and-6)-carboxy-2′,7′-dichlorodihydrofluorescein diacetate (carboxy-H_2_DCFDA) (Life Technologies, USA). Fresh brain cortex tissue was homogenized (1/5; w/v) in Locke buffer (154 mM NaCl, 5.6 mM KCl, 1.0 mM MgCl_2_, 2.3 mM CaCl_2_, 8.6 mM HEPES, 5.6 mM glucose, 0.1 mM glycine, pH 7.4). Homogenate were than diluted 10 times and divided into two parts. First were incubated in dark (15 min, 25 °C) with 10 μM fluorescent probe second with corresponding volume of DMSO. 200 μL of solution were used to measure the fluorescence intensity at an excitation wavelength of 485 nm and emission wavelength of 515 nm (FLUOstar OMEGA, BMG Labtech, USA). Fluorescence intensity were directly proportional to the intracellular ROS. The results are presented as fluorescence intensity/mg of protein.

### GSSG/GSH Determination

Oxidized and reduced glutathione were measured using Quantification kit for oxidized and reduced glutathione (Sigma-Aldrich, Germany) according to the manufacturer’s protocol. Briefly, 100 mg of tissue were homogenized in 0.75 ml of 5 % SSA and centrifuged at 8000×*g*, 10 min, 4 °C. The supernatant was transferred to a new tube and SSA concentration was reduced to 0.5 % by addition of ddH_2_O. 40 μl of samples and GSH and GSSG standards were added into microplate. For determination of GSSG only the “masking reagent” binding GSH was added to part of the samples. After incubation with buffer solution 1 h, 37 °C the 20 μl of coenzyme solution and enzyme solution was added to each well. GSSG and total glutathione (GSH + GSSG) were determined detected spectrophotometrically at 415 nm. The GSH was quantified using following equation: GSH = total glutathione − GSSG × 2.

### Western Blot

The CARNS-1 protein content in rat brain cortex was determined by Western blotting. The brain tissue were homogenized in Triton Lysis Buffer (20 mM Tris pH 6.8, 137 mM NaCl, 2 mM EDTA, 1 % Triton X-100, 0.5 mM DTT, 1 mM PMSF) containing Protease Inhibitor Coctail (Sigma-Aldrich, Germany) and Phosphatase Inhibitor Cocktail (Sigma-Aldrich, Germany). The homogenate was centrifuged for 20 min at 12,000×*g*, 4 °C. Equal amounts of protein (30 μg) were separated on 10 % SDS–polyacrylamide gel and transferred onto nitrocellulose membrane. The membranes were incubated over-night with an anti-CARNS1 antibody (1:400; Proteintech, USA), washed, incubated with HRP-conjugated anti-rabbit IgG (1:8000; Sigma-Aldrich, Germany), and developed using West-Pico Chemiluminescence Substrate (Pierce, Rockford, USA). After stripping, the blots were incubated with an anti-GAPDH antibody for 1 h (1:10,000; Sigma-Aldrich, Germany) and developed as described above. The chemiluminescent signal acquisition and densitometry analysis were conducted using the G-Box system (SynGene, USA) and GeneTools software (SynGene, USA), respectively. Results were presented as % of control.

### Behavioral Analysis

**Fig. 1:**
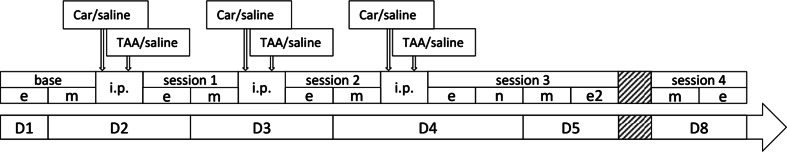
Schematic plan of behavioral sessions and TAA/Car/saline i.p. injections during several days (D). More information in the text

A total of 70 animals were used. These were injected as described above and represented TAA (n = 20), TAA-Car (n = 20), Car (n = 15), and control (n = 15) groups. Behavioral experiments were divided into sessions before (baseline) and after intraperitoneal injections of TAA and/or Car or saline (Fig. 1). Sessions were divided into: evening (e), 3 h post last injection (p.i.), and morning (m), 20 h p.i. parts. Session 3 had additional night (n), 9 h p.i., and evening (e2), 27 h p.i., parts. Session 4 was performed 7 days after the first injection. The experimenters were blind to the animals’ treatments. Before the experiments, the rats were handled 3 times for 2 min.

The impact of TAA and TAA-Car on six reflexes was analyzed during sessions 1 m, 2 m, 3 e, 3 n, 3 m, and 4 m. *Righting reflex* (RR) [30–32], where rats were grabbed by the scruff and the base of the tail, flipped on their back, and put in a supine position into the test cage without bedding and released. The time required to achieve prone posture, with all four paws on the floor, was measured with maximal time of 3 s. Other reflexes [30] were scored with a scale of 2-1-0, where 2 is a normal, healthy state, 1 signifies a presence of a response but different from normal, and 0 is a lack of a response/reflex. *Startle reflex*, analogous to scatter reflex [33, 34], experimenter clapped his hands above the cage and the startle reaction to this sudden stimulus was scored, 2—strong startle reaction, 1—rat reacts to the noise but is not startled, 0—no reaction. *Postural reflex* (PR) [30, 35], test cage was held by the experimenter and vigorously moved in vertical and horizontal motion. The ability of the rat to maintain balance was scored as: 2—rat has the legs extended, with four paws almost all the time on the floor and uses tail to balance, with maintained upright position, 1—rat struggles to maintain balance or briefly loses balance, 0—rat cannot maintain balance and rolls. *Whisker-orienting reflex* [30], freely moving rats’ whiskers were stimulated by gentle stroking with a rod. Response to the stimulus was scored, 2—head twisting, stopping whiskers’ moves, 1—only moving whiskers stopped but no head motion, 0—rat does not move whiskers at all, indifferent to stimuli. *Ear twitch reflex* [30], rat’s ear was stimulated by a rod and the response was scored, 2—rat pulls back ear or twitches it and/or moves the head, 1—rat responds very briefly by moving ear, 0—no ear movement observed. *Eye blink reflex* [30, 36], the rat’s eye was gently poked by a water-moistened swab and reaction was scored, 2—the eye is wide open and is closed then the swap is near or touches it, 1—the eye is half open but eyelids close when touched, no preliminary eye shutting when swab is close, 0—eyes are permanently opened or closed, minimal or none eyelid movement when touched.

*Open field* (OF) was performed during sessions: Be, 1e, 2e, 3e, 3n, 3e2, and 4e. OF dimensions were 55 × 55 × 50 cm. Rats were gently placed in the corner of the OF floor facing the walls. Recording lasted for 5 min. After each trial, the apparatus was cleaned with 10 % ethanol solution. Animal behavior was recorded with Basler acA1300-60 GigE (Bassler AG, Germany) camera and scored using Ethovision XT 10 (Noldus Information Technology, Netherlands). Mean velocity (in cm/s) and total distance moved (cm) were measured [37–39]. *Rotarod* (Accelerating Rota-Rod 7750, TSE systems, Germany) test was performed on Be, 1e, 2e, 3e, 3n, 3e2, and 4e sessions. The test was preceded by habituation to the device by placing the rat on a stationary cylinder for 30 s and thereafter for 2 min with a constant low speed rotation (4 rpm). The animals that fell from the rod were placed again on it until they were able to stay for 60 s. This procedure was performed for 2 days prior to the test. After this the animals were tested in accelerating conditions. The cylinder accelerated from 4 to 40 rpm in 300 s. The time of the trial was scored when the rat fell from the cylinder, spun with the cylinder three times consecutively without walking or reached 500 s without falling. The device was cleaned with 10 % ethanol solution between animals [40]. *Grip Test* (Bioseb BP, In Vivo Research Instruments, France) was done in sessions Bm, 1 m, 2 m, 3n, 3 m and 4 m. To measure the forepaw grip strength of the rat the animal was held by its trunk and the base of the tail. Then it was guided onto a metal grid (0.5 cm square opening) attached to a force transducer and encouraged to grab it with forepaws only. Then it was steadily pulled backwards until it lost hold of the grid. Three measurements in newtons per rat were taken with at least 1 min of interval between trials to let the animal rest [41, 42].

### Protein Determination

Protein content was determined using BCA Protein Assay Kit (Thermo-Scientific, USA) according to manufacturer’s protocol.

### Statistical Analysis

The results were expressed as mean ± SD or SEM (for behavioral testing, Fig. 7). Differences among experimental groups were evaluated using Mann–Whitney test or one-way ANOVA analysis of variance with post hoc Bonferroni’s test using Prism 3.0 software, *p* value lower than 0.05 was considered statistically significant.

## Results

Carnosine (Car) concentration in brain cortex homogenate from TAA rats was decreased by ~15 % (Table 1). His administration increased Car concentration in brain homogenate from control rats by ~25 %, and abolished the reduction of Car content in TAA rats (Table 1). Otherwise, an i.p. administration of Car did not change its concentration measured in the brain homogenates, nor concentration of His (Table 1). The level of Car measured in the plasma of all experimental groups was under the detection limit (data not shown).

**Table 1:** Effects of i.p. administration of histidine (His) and carnosine (Car) to control or TAA-treated rats on the His and Car concentration in rat brain cortex

	Control	TAA	His	Car	TAA + His	TAA + Car
His (µM)	425.5 ± 102.6	528.9 ± 127.7	328.3 ± 80.0	378.2 ± 82.3	334.8 ± 90.7	512.5 ± 118.2
Car (µM)	28.6 ± 1.9	24.2 ± 0.8*	35. 3 ± 2.0^#^	31.4 ± 2.1	38.5 ± 2.7^#^	30.7 ± 2.9

Results are presented as mean ± SD (n = 6). Statistical significance was calculated using one-way ANOVA analysis of variance with post hoc Bonferroni’s test

* *p* < 0.05 versus control

^#^ *p* < 0.05 versus TAA

**Fig. 2:**
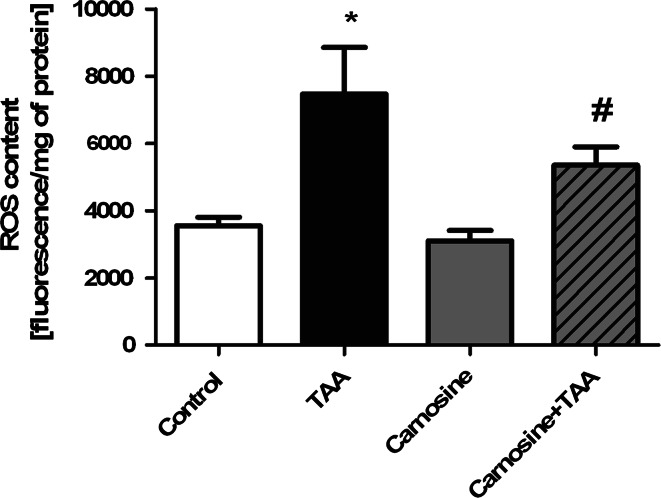
The effect of carnosine administration on ROS concentration in brain cortex of control and TAA rats. Results are mean ± SD (n = 4–6); * *p* < 0.01 versus control, ^#^ *p* < 0.05 versus control and TAA

**Fig. 3:**
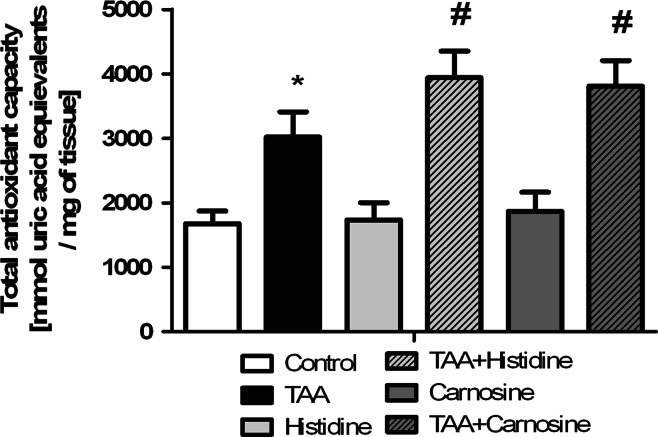
The effect of systemic administration of His or Car on the total antioxidant capacity (TAC) measured in the rat brain cortex of control and TAA rats. Results are mean ± SD, (n = 6); * indicates *p* < 0.05 versus control, ^#^ *p* < 0.05 versus control and TAA

**Fig. 4:**
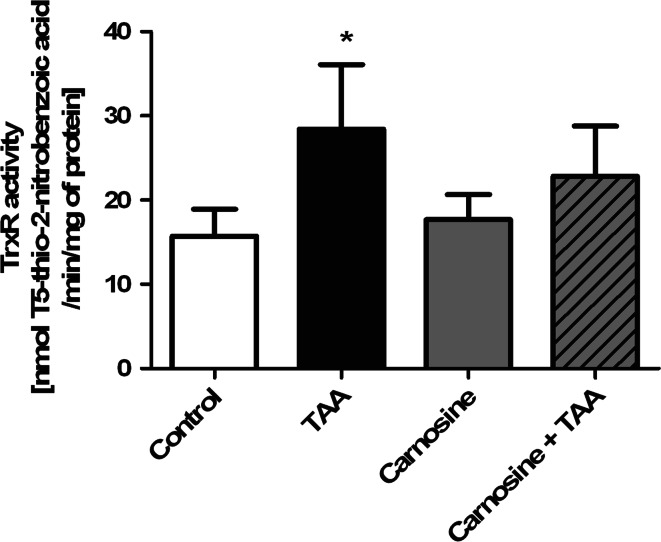
Effect of systemic administration of carnosine (Car) on thioredoxin reductase (TrxR) activity in brain cortex of control and TAA rats. Results are mean values ± SD (n = 5–6); * indicates *p* < 0.05 versus control

As was shown previously, TAA treatment increased almost twice the ROS production in rat brain cortex. Administration of Car significantly reduced TAA-induced ROS formation and tended to decrease ROS content in control rats (Fig. 2). In agreement with earlier reported data [26], the total antioxidant capacity (TAC) in brain homogenates from TAA rats was increased by ~50 %. While administration of His or Car did not affect TAC in control brains, each potentiated TAC in TAA -affected brain (Fig. 3). TrxR activity in brain homogenates of TAA rats was increased by ~75 %, and Car treatment significantly reduced the increase (Fig. 4).

**Fig. 5:**
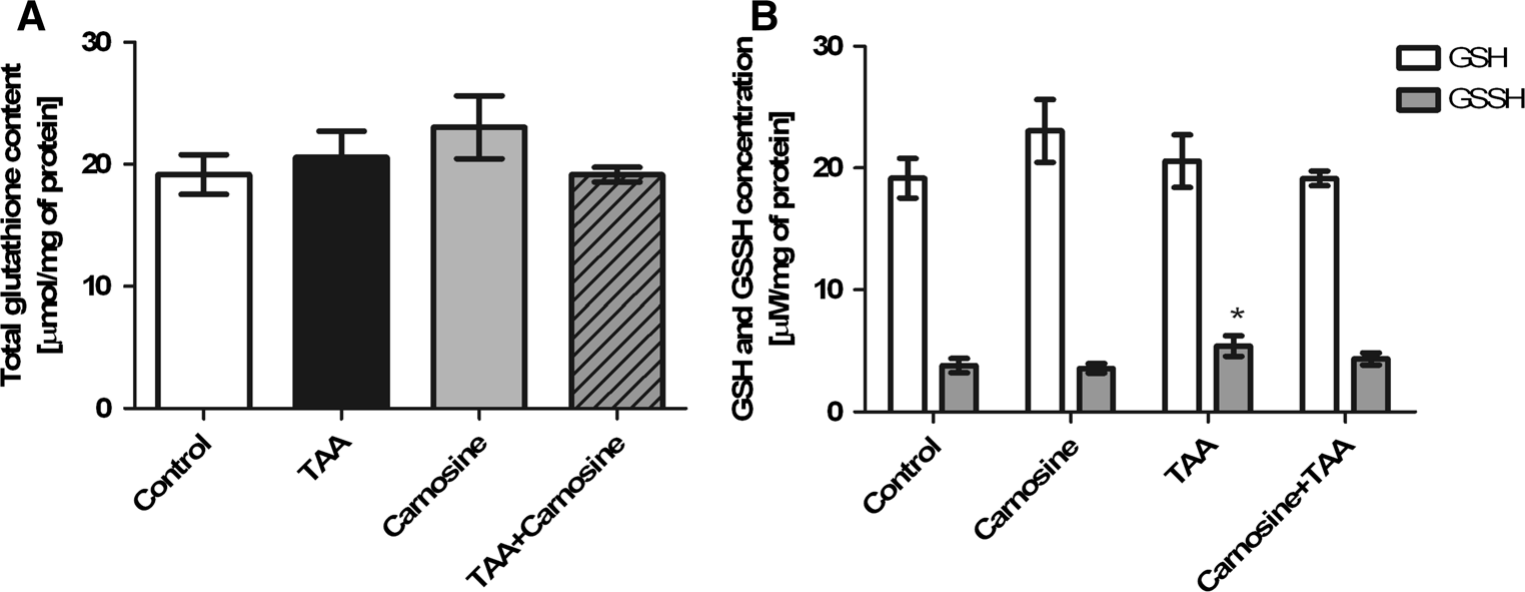
The effect of carnosine administration on total glutathione (**a**) and reduced (GSH) or oxidized (GSSG) glutathione concentration (**b**) in rat brain cortex of control and TAA rats (n = 4). Results are mean values ± SD; * indicates *p* < 0.05 versus control

Although total glutathione content did not significantly differ between all groups (Fig. 5a), an increase of oxidized form of glutathione (GSSG) was observed in TAA homogenates (Fig. 5b). The GSSG/GSH ratio was elevated by ~35 % in TAA group of rats, and Car reversed this change (Fig. 5b). Administration of Car resulted in reduction of the GSSG/GSH ratio by ~25 % in control rats, proving its protective effect against glutathione oxidation.

**Fig. 6:**
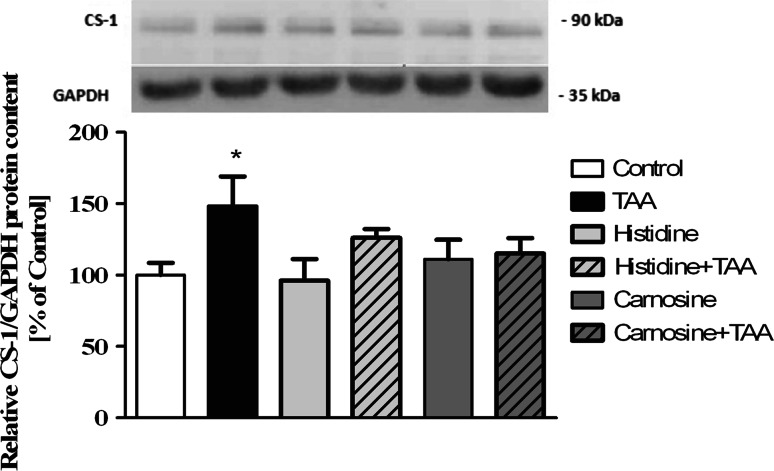
The expression of carnosine synthase-1 (CARNS-1) protein in cerebral cortex of control and TAA rats after administration of His or Car (**a**). Representative electropherogram (**b**). Values in each group are mean ± SD (n = 4); * indicates *p* < 0.05 versus control

The protein expression of carnosine synthase-1 (CARNS-1), the enzyme converting His and β-alanine to Car, was increased in TAA rat brain (Fig. 6). Systemic administration of Car or His did not influence the CARS-1 protein content (Fig. 6).

**Fig. 7:**
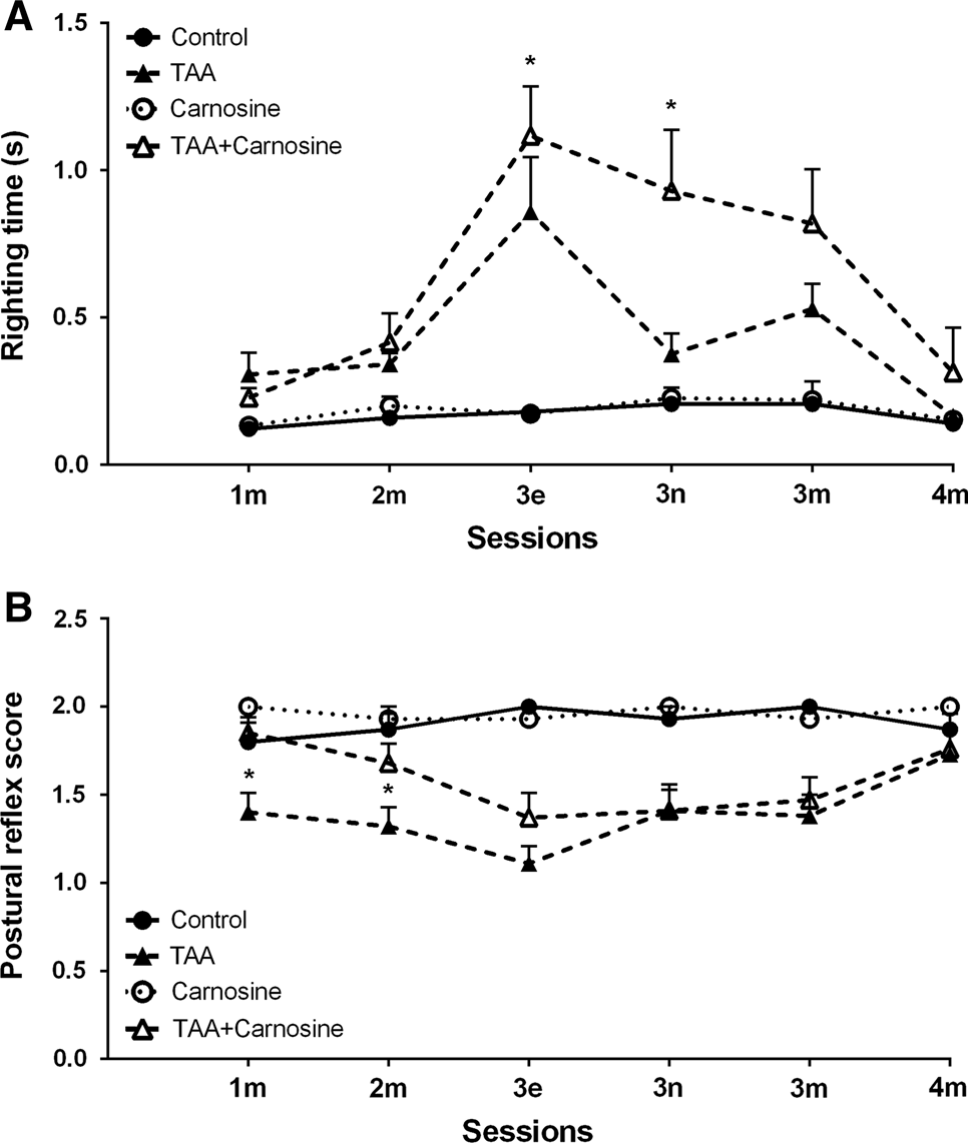
The effect of Car injection on righting (RR, **a**) and postural reflexes (PR, **b**) following TAA treatment; * *p* < 0.05 for the difference between TAA and TAA-Car groups. TAA-Car animals took shorter times to right themselves up than TAA rats (**a**); TAA-Car animals scored higher during PR testing than TAA rats (**b**). More information regarding sessions (see also Fig. 1) and differences between TAA groups and corresponding control groups is in the text

For RR (Fig. 7a), there was a difference between TAA and TAA-Car animals during sessions 3e and 3n (*p* < 0.05 for both, Mann–Whitney) with TAA animals taking longer times to revert to the upright position than TAA-Car rats. Also TAA animals differed form control rats during 1 m (*p* < 0.05), 2 m (*p* < 0.01), 3e (*p* < 0.001), 3n (*p* < 0.001), and 3 m (*p* < 0.001) sessions, while TAA-Car rats also took longer times than Car animals to achieve prone posture at sessions 2 m (*p* < 0,05), 3e (*p* < 0.001), and 3 m (*p* < 0.01, all Mann–Whitney tests).

For PR (Fig. 7b), there was a difference between TAA and TAA-Car animals during sessions 1 m and 2 m (*p* < 0.05 for both, Mann–Whitney) with TAA-Car animals reaching higher scores than TAA rats. Also TAA animals differed form control rats during 1 m (*p* < 0.05), 2 m (*p* < 0.01), 3e (*p* < 0.001), 3n (*p* < 0.01), and 3 m (*p* < 0.001) sessions, while TAA-Car rats also had lower scores than Car animals during posture testing at sessions 3e (*p* < 0.01), 3n (*p* < 0.001), 3 m (*p* < 0.01), and 4 m (*p* < 0.05, all Mann–Whitney tests).

Also, in several other behaviors TAA-Car rats showed results closer to control values than TAA rats (not shown). For remaining 4 reflexes, analyzed in 6 sessions (24 measurements), TAA-Car animals had higher scores 17 times, while equal scores were noted 5 times. In OF, at 1e session, TAA-Car rats covered longer distance (692 ± 142 cm) than TAA animals (523 ± 77 cm), also with similar results during 3n session (675 ± 85, 531 ± 104, respectively). In Rotarod test, where TAA-Car animals (106.9 ± 9.0 rpm) stayed longer on the rod than TAA rats (88.8 ± 7.5 rpm) during 1e session. Finally, for Grip test analysis, TAA-Car animals displayed results which were closer to values represented by control groups, during 1 m session (TAA-Car rats, 7.0 ± 0.5 N; TAA rats, 7.2 ± 0.3 N). Finally, the weight of TAA-Car rats (235.1 ± 5.1) was higher than TAA animals (220 ± 7.1) following the experiments. All these differences were weak and did not prove significant, however the differences between TAA and control groups were significant in case of open field measurements, startle reflex index, Rotarod performance, and animals’ weight.

## Discussion

Pathogenesis of HE is associated with oxidative stress (OS) [8, 43], which mainly results from inefficient clearance of ROS by natural defense systems composed of enzymatic and non-enzymatic anti-oxidants [44, 45]. HE induces ONS by complex interrelated mechanisms including, among others, mitochondrial dysfunction and neuro-inflammation [3, 5, 46, 47]. Therefore, experimental attempts at designing novel therapeutic strategies included among many others [48] the use of antioxidants: N-acetylcysteine [49], His [20, 25], taurine [50]. Of these, N-acetylcysteine and His also reduced incidence of cerebral edema, and prevented the onset and progression of HE in animal models of acute or chronic liver failure [20, 51]. In the present study, we tested the therapeutic potential of systematically administered of one other potential antioxidant, β-alanyl-l-histidine (Car) in a well-established TAA model reproducing cerebral metabolic changes and symptoms of acute HE [28]. Car is a major constituent of excitable tissues, brain and skeletal muscles [52]. However, the biological role of Car and the mechanisms underlying its action are s still a mystery, although several theories have been proposed. Car is thought to act as a buffer, neutralizing lactic acid produced in a working muscle [19], mainly due to its abundance and pKa close to physiological pH. Car also acts as a radical scavenger in muscle and brain [53], and as a neurotransmitter in the olfactory system [10]. Also, together with a related structurally peptide, homocarnosine, Car may act as a γ-aminobutyrate reservoir availability control for this neurotransmitter [54]. As mentioned in the introductory paragraph, Car has been shown to ameliorate OS associated with cerebral ischemia, stroke or subarachnoid hemorrhage [11–13]. In this study, we demonstrated that intraperitoneal administration of Car decreased ROS level and increased total antioxidant capacity (TAC) by partial improvement of thioredoxine- and GSH/GSSG antioxidant systems. These findings support our hypothesis that Car exerts its anti-oxidative activity in HE-affected brain and as such adds to the list of antioxidants that may be considered as therapeutic agents in HE. The therapeutic potential of Car found support in behavioral studies. HE impairs cognitive and locomotor activity in humans [55–57] and experimental animal models [17, 58]. Here we demonstrated that the TAA-induced aspects of motor deficit that are typical of HE have been largely attenuated by intraperitoneal administration of Car. Collectively, this report adds HE to the list of brain pathologies in which Car may exert neuroprotection by ameliorating OS. As mentioned in the introductory paragraph, Car remains in a reversible precursor-product relationship with His. In different experimental settings imitating HE pathology, both in vitro and in vivo exogenously added His exerted neuroprotection. In a similar TAA-induced rat model of HE, His counteracted mitochondrial permeability transition (mPT) and brain edema associated with acute HE [20]. The beneficial effects of His appeared to be related to the inhibition of the transport to mitochondria of toxic concentrations of Gln, as a consequence of ammonia detoxification [59]. However direct anti-oxidative properties have been reported as well [23], and for references see [24]. Irrespective of the mechanism considered, effective reduction of the oxidative-nitrosative stress (ONS) has been observed. This raised a question whether and in what degree, the metabolism of His to Car, may be involved in the neuroprotective action of His. The present study partly confirms the latter possibility: His administered i.p. increased Car concentration in control and TAA rat homogenates, indirectly suggesting effective synthesis of Car dipeptide in this experimental settings.

Carnosine synthetase-1 [CARNS-1; (EC 6.3.2.11)] is the main enzyme directly catalyzing formation of Car [52]. In our study the protein expression of CARNS-1 was increased in TAA rats, a finding which is discrepant with the decrease of Car concentration noted in the same conditions (Table 1). While there is no simple explanation of this discrepancy, two mutually not exclusive effects of HE may be considered: (i) increased Car degradation and (ii) induction of as yet undefined factors which inhibit carnosine synthase-1, resulting in abrogation of the effect of increased expression. Clearly, the significance of increased enzyme expression will have to be validated by direct activity tests. The absence of changes in β-alanine content (data not shown) is not critical for the interpretation of active metabolism of His in our study. Considering massive His content in the rat cortex homogenate, and its unlimited availability, β-Ala is unlikely to be a rate-limiting factor in His conversion to Car. One interesting point bespeaking the strength of the effect of Car in our study is that the protective effect was noted at a low dose (100 mg/kg), whereas in many other studies significant effects of Car are observed at 500–1000 mg/kg [12, 60].

The use of the lower dose of Car was probably the reason for only subtle and transient behavioral differences observed, the most pronounced being RR and PR. These behaviors were regarded as affected in rodent models of encephalopathy [33, 36] or ammonia toxicity [35]. RR and PR reflect complex underlying functions incorporating components of wakefulness/consciousness, motor abilities, effective balance and vestibular system performance, control of visual and somatosensory inputs as well as motivation [61–63]. RR and PR are also related as maintaining proper balance and posture are both vestibular system reflexes which are regulated by the vestibular nuclei as a result of inputs coming from the vestibular sensors, the cortex, the cerebellum, ocular stimuli and neck proprioception. [64] Future investigations should determine, which of these behavioral and anatomical elements are mostly affected by Car attenuating effects of TAA treatment.
